# Improving the Viability of Probiotics under Harsh Conditions by the Formation of Biofilm on Electrospun Nanofiber Mat

**DOI:** 10.3390/foods11091203

**Published:** 2022-04-21

**Authors:** Jiao Shi, Shu-Fang Li, Kun Feng, Shuang-Yan Han, Teng-Gen Hu, Hong Wu

**Affiliations:** 1 Guangdong Province Key Laboratory for Green Processing of Natural Products and Product Safety, School of Food Science and Engineering, South China University of Technology, Guangzhou 510640, China; shijiao0404@163.com (J.S.); lishufang1123@163.com (S.-F.L.); fengkun_89@163.com (K.F.); 2 School of Biology and Biological Engineering, South China University of Technology, Guangzhou 510640, China; 3 Sericultural & Agri-Food Research Institute, Guangdong Academy of Agricultural Sciences/Key Laboratory of Functional Foods, Ministry of Agriculture/Guangdong Key Laboratory of Agricultural Products Processing, Guangzhou 510630, China; hu.tenggen@foxmail.com

**Keywords:** electrospun nanofiber mat, biofilm, probiotics, viability, *luxS* gene

## Abstract

For improving probiotics’ survivability under harsh conditions, this study used *Lactiplantibacillus plantarum* GIM1.648 as a model microorganism to investigate its ability to produce biofilms on electrospun ethyl cellulose nanofiber mats. SEM observations confirmed that biofilm was successfully formed on the nanofibers, with the latter being an excellent scaffold material. The optimal cultivation conditions for biofilm formation were MRS medium without Tween 80, a culture time of 36 h, a temperature of 30 °C, a pH of 6.5, and an inoculum concentration of 1% (*v/v*). The sessile cells in the biofilm exhibited improved gastrointestinal and thermal tolerance compared to the planktonic cells. Additionally, the RT-qPCR assay indicated that the *luxS* gene played a crucial role in biofilm formation, with its relative expression level being 8.7-fold higher compared to the planktonic cells. In conclusion, biofilm formation on electrospun nanofiber mat has great potential for improving the viability of probiotic cells under harsh conditions.

## 1. Introduction

According to the FAO/WHO, probiotics refer to “live microorganisms that, when administered in adequate amounts, confer a health benefit on the host”. Due to their beneficial effects, probiotics are being increasingly applied nowadays to prevent and treat inflammatory bowel disease and irritable bowel syndrome, as well as other human diseases [1]. Although a great number of probiotic foods have emerged in recent years, their consumption remains challenging as they are quite susceptible to the harsh conditions under which they are processed and delivered to the gastrointestinal (GI) tract. Presently, various microencapsulation techniques, namely extrusion, freeze drying, spray drying, emulsification, and electrospinning, can be applied to protect probiotics against harsh conditions [2,3,4], but compared to these methods, enhancing the self-resistance of probiotics during the production process represents a much simpler strategy, which has been drawing the attention of researchers [5].

Biofilms, often made up of polysaccharides, DNA, and proteins, are highly complex but organized collections of microorganisms that are often fixed onto a carrier surface [6]. Even though biofilms from some pathogenic bacteria can lead to undesirable effects, there are nevertheless many clear examples in which the high biomass density and resistance of biofilms have resulted in useful applications, such as for the production of chemicals, fermented foods, and anti-microbial compounds. For example, it was reported that biofilms from *Lactiplantibacillus plantarum* (*L. plantarum*), formed on a nanofiber mat, could be employed as a starter culture for producing yogurt that had higher viability during shelf life [7]. It was also found that *L. rhamnosus* GG, encapsulated in biofilms, displayed better stability at both high and low temperatures compared to a similarly-encapsulated planktonic strain [8]. Some studies have further reported significantly improved survivability of lactic acid bacteria [9] and *L. plantarum* LIP-1 strains [10] when they were present within biofilms during gastrointestinal digestion or in freeze-drying environments, compared to their planktonic state. Hence, producing probiotic biofilms through which their resistance to unfavorable environments can be improved could be a useful approach.

Generally, a broad range of materials, including inorganic and organic compounds, can be used as a support for microbial adhesion. Compared to inorganic materials (i.e., stainless steel and ceramics), organic ones are likely to be more advantageous as their porosity can be tailored according to the needs of the target organism [11]. At present, electrospun fiber mats, widely used in the tissue engineering field, can be used to model a natural extracellular matrix so as to improve microbial adhesion and growth on the fibers. In this respect, electrospun fiber mats have, therefore, been considered as suitable materials for the producing biofilms [12]. Biofilm formation is a very complex process that is influenced by several variables, such as cultivation conditions (medium, pH, temperature, etc.), scaffold properties (surface roughness, hydrophilicity, charge, etc.), and types of strains (cell structure, gram-negative or gram-positive, age, species, etc.) [13]. Of these, the type of medium as well as the pH and temperature of the culture are some of the most important factors, as their effects are not only variable but may also differ between species [14].

In order to increase biofilm formation to improve the ability of probiotics to resist harsh conditions, *L. plantarum* GIM1.648 was selected as a model probiotic, and for the first time, its capacity to form biofilms on electrospun ethyl cellulose nanofiber mats was studied. In addition, the effects of environmental conditions (medium and culture conditions) on biofilm formation were investigated, along with the protective effects of the biofilms against gastrointestinal digestion and heat treatment. Eventually, real time quantitative polymerase chain reaction (RT-qPCR) assays were used to compare the expression of the *luxS* gene before and after biofilm formation in order to better understand the relationship between the gene expression and biofilm formation.

## 2. Materials and Methods

### 2.1. Materials

Ethyl cellulose (EC) was purchased from Aladdin Biochemical Technology Co., Ltd. (Shanghai, China), while pepsin (3000 U/mg) and porcine trypsin (250 U/mg) were obtained from Aladdin biological technology Co., Ltd. (Shanghai, China). Acetic acid was purchased from Sinopharm Chemical Reagent Co., Ltd. (Shanghai, China). DeMan-Rogosa-Sharpe (MRS) medium and bile salts were obtained from Guangdong Huankai Microorganism Technology Co., Ltd (Guangzhou, China). The selected strain for this study was *L. plantarum* GIM1.648, which was first activated in MRS broth. To ensure its viability, the strain was then sub-cultured twice and stored at −80 °C until it was required for use.

### 2.2. Preparation of Nanofiber Mat

All of the nanofiber mats used were prepared by an electrospinning method using a syringe pump (NE-300, New Era Pump Systems Inc., Farmingdale, NY, USA) and a voltage power supply (ESS0P-5W/DAM, Gamma High Voltage Research, Ormond Beach, FL, USA). For preparing the electrospinning solution (50%, *w/v*), EC was dissolved in acetic acid before being vigorously stirred (1000 rpm) by a magnetic stirrer for 1 h at room temperature. For electrospinning, a grounded collecting plate covered by a piece of aluminum foil was employed as the collector for the fiber deposition. The applied voltage was 15 kV. The distance between the needle tip and collector was 18 cm. The feed rate of solution was controlled at 0.5 mL/h by a syringe pump. The electrospinning process was conducted in a home-made Plexiglas box, where the temperature (25 ± 1 °C) and relative humidity (33 ± 2%) were controlled by the air conditioner. To remove any remaining solvents, the resulting nanofiber mats were dried for 12 h at 50 °C in a vacuum oven.

### 2.3. Characterization of Nanofiber Mats

The hydrophobicity of the materials was characterized by contact angle measurements. Using distilled water as the test liquid, the contact angle of a drop of water (4 μL) on a mat was measured by the sessile drop method at room temperature, with the help of an Attension Contact Angle Meter (Theta Flex, Biolin Scientific Instrument, Gothenburg, Sweden). In this way, each mat was measured at different positions for six times. Eventually, the angle between the tangent line at the drop boundary and the baseline of the drop was measured.

A three-dimensional (3D) optical profiler (UP Dual Model, RTEC, America) provided a quantitative analysis of the material’s surface roughness. The nanomaterial was first prepared by electrospinning before being directly attached to a glass slide (2 cm × 5 cm). The light source was set to white light, and the scanning was performed in confocal mode with a BF-20x lens. The 3D profile information map and average surface roughness of the sample were further obtained using Gwyddion software.

### 2.4. Biofilm Formation and Characterization

Round samples, of 3 cm in diameter, were prepared before being kept in a laminar flow biological safety cabinet for a 1-h sterilization under ultraviolet light. The sterilized EC mat was subsequently used as a scaffold for biofilm formation by *L. plantarum* GIM1.648. For this purpose, a sterile piece of mat was kept in a glass petri dish, to which 3 mL of MRS medium inoculated with 2% (*v/v*) of a *L. plantarum* GIM1.648 culture had been added. The petri dishes were then sealed with parafilm to avoid evaporation prior to cultivation for 24 h at 37 °C and under static conditions to obtain single-species biofilms. As a control, the pure fiber mat cultured in sterile MRS without the addition of bacterial suspension was treated with a similar method.

After the cultivation process, samples of mats with biofilms were rinsed gently with sterile saline solution (0.85%, *w/v*) three times in order to remove planktonic cells. This was followed by sample dehydration for at least 48 h at room temperature before characterization by field emission scanning electron microscopy (SEM; SU 5000, Hitachi, Japan) to observe the stage of biofilm formation. In this case, after sputter coating with platinum for 1 min using a sputter coater (MC1000, Hitachi, Japan), at least three different parts of the mat were selected to observe the biofilms under a magnification of 4000× and an accelerating voltage of 5 kV. The morphology of the EC nanofiber mat without biofilm was also observed by SEM.

### 2.5. Quantitation of Sessile Cells in Biofilms

The number of sessile cells in the biofilm was quantitatively determined by the plate counting method. Measuring colony-forming units (CFU) has been shown to be a simple and appropriate method for studying biofilms produced by different types of bacteria such as Staphylococci (*S. aureus* and *S. epidermidis*) and *Escherichia coli*, and thus, in this study, the quantitation of sessile cells in biofilm was performed according to Hu’s method of CFU enumeration with some modifications [7]. Briefly, after cultivation, the biofilm formed on the nanofiber mat was washed, as described before, to remove planktonic cells. Ultrasounds (150 W, 25 °C, 10 min) were subsequently used to detach the sessile cells from the biofilm. The resulting cell suspension was then serially diluted 10-fold, with 100 μL from the dilutions plated in triplicate onto MRS agar. After incubation at 37 °C for 48 h, the number of CFU was eventually counted.

### 2.6. Impact of Growth Conditions on the Formation of Biofilms

The influence of MRS on the ability of *L. plantarum* GIM1.648 to produce biofilms was compared with that of modified trypticase soy broth (mTSB), supplemented with (2%, *w/v*) of proteose peptone medium. For this purpose, EC nanofiber mats were used as a scaffold material, as described in Section 2.4. As a control, the pure fiber mat cultured in sterile MRS without the addition of bacterial suspension was treated in a similar method. Furthermore, the effects of Tween 80 (MRS with or without Tween 80) on biofilm formation was investigated. In both cases, the microbial culture for biofilm production and the CFU enumeration were carried out as described in Section 2.4 and Section 2.5, respectively.

In addition, to determine the influence of culture conditions (cultivation time, culture temperature, pH, and inoculum concentration) on biofilm formation, the growth parameters were varied as follows: cultivation time was changed from 12 h to 72 h, culture temperatures of 25 °C, 30 °C, and 37 °C were used; the pH varied from 4.5 to 6.5; the inoculum concentration varied between 0.1% and 3% (*v/v*). For these experiments mentioned above, the optimized medium was selected, with all the other experimental conditions remaining unchanged as described in Section 2.4. After changing each parameter, the number of CFU was determined as described in Section 2.5.

### 2.7. In Vitro Gastrointestinal Tolerance of Biofilm

For preparing the simulated gastric fluid (SGF), the pH of 0.85% (*w/v*) sodium chloride solution was first adjusted to 3 with 1 M HCl before adding pepsin (final concentration of 3 g/L). For the simulated intestinal fluid (SIF), after adjusting the pH of 0.85% (*w/v*) saline water containing 0.3% (*w/v*) bile salts, trypsin was added (final concentration of 1 g/L). Both fluids were sterilized using 0.22-μm pore size membranes before digestion experiments.

For planktonic cells (control), 3 mL of bacterial culture was directly centrifuged (6000 rpm, 5 min). For biofilm growth modes, after removing culture supernatants, the biofilms were carefully rinsed with saline water so that only sessile cells were retained. Both biofilm and planktonic pellets were subsequently incubated for 2 h with 3 mL of SGF in a shaker incubator (37 °C, 100 rpm). When incubation was over, sterile saline water was used to wash the samples three times before transferring them to SIF (37 °C, 100 rpm, 3 h) for digestion. Eventually, all samples were resuspended in 0.85% (*w/v*) saline water for viable cell counts, with ultrasounds (150 w, 25 °C, 14 min) required in the case of biofilms to get the cells into the saline water. Viable cell counts for both growth modes were obtained by drop plating serially-diluted samples on MRS agar. The survival rate of cells after simulated gastrointestinal digestion was then calculated according to the following Equation (1):

Survival rate (%) = N/N_0_ × 100, (1)

where N is the number of viable cells in both modes after simulated gastrointestinal digestion (log CFU/g), and N_0_ refers to the number of viable cells in both modes before simulated gastrointestinal digestion (1og CFU/g).

### 2.8. Thermal Tolerance of Biofilm

The heat stability of sessile and planktonic cells, the latter being the control, was determined as described previously with some modifications [15]. For the planktonic growth mode, 3 mL of bacterial culture was directly centrifuged (6000 rpm, 5 min). In the case of the biofilm growth mode, the removal of culture supernatants was followed by careful rinsing of the biofilms with saline water so that only the adherent bacteria were retained. Both biofilm and planktonic pellets were then cultivated in 3 mL of sterile MRS medium in a water bath for 30 min at the following temperatures: 55 °C, 60 °C, 65 °C, and 70 °C. After heat treatment, cells in both growth modes were resuspended in sterile saline water for viable cell counts, as described in Section 2.7.

### 2.9. RT-qPCR Assay

Primer Premier 5.0 software was first employed to design primers for the following genes: *luxS*—(F: 5′-TTAGATCATACCAAGGTTTTAGCAC; R: 5′-TGTAAGCCCGCCGTATCA), *16S rRNA*—(F: 5′-CTGGTCTGTAACTGACGCTGAG; R: 5′-CTCCAACACTTAGCATTCATCG).

The planktonic cells were used as the control group. They were cultivated in a flask, and the detailed conditions were MRS medium, a culture time of 36 h, a temperature of 37 °C, original pH, and an inoculum concentration of 1% (*v/v*), and RT-qPCR assay was carried out to identify gene expression (*luxS*) of sessile cells, which were cultivated under optimal environmental conditions based on the results of biofilm studies. Total RNA was then extracted using UNIQ-10 pillar Trizol total RNA extraction kit (Shanghai Sangon Biological Engineering Technology & Service Co., Ltd., Shanghai, China) according to the manufacturer’s instructions. This was followed by reverse transcription with the Maxima Reverse Transcriptase (Code No: EP0743, Thermo Scientific, Waltham, MA, USA) prior to quantitative PCR on an ABI Stepone plus system (Applied Biosystems, Foster, Waltham, MA, USA) using the 2X SYBR Green qPCR Master Mix (High Rox, B639273, BBI, ABI). To determine levels of gene expression, ct values of the *luxS* gene were normalized, using the *16S rRNA* gene as a reference. Relative gene expression was then analyzed by the 2^−ΔΔCt^ method.

### 2.10. Statistical Analysis

Experiments were carried out with three replicates, with the data provided as the mean ± standard deviation. Using SPSS Statistics Software (v.22.0), means were compared by one-way analysis of variance (ANOVA) and assumed to be statistically significant at *p* < 0.05.

## 3. Results and Discussion

### 3.1. Biofilm Formation on the EC Nanofiber Mat

**Figure 1:**
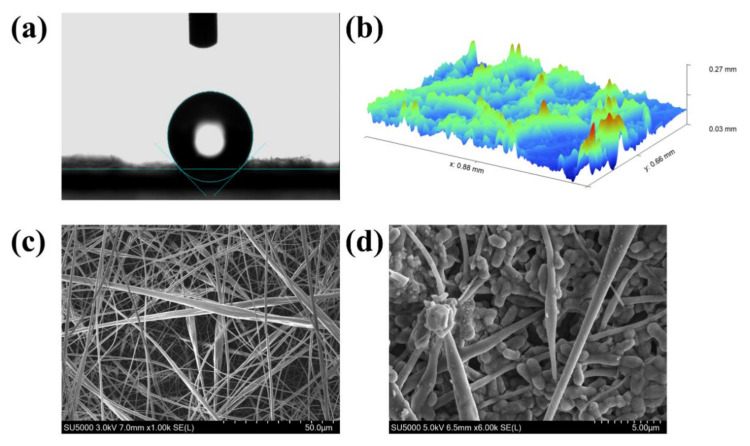
(**a**) Water contact angle; (**b**) 3D profile information map; (**c**) SEM image of ethyl cellulose (EC) nanofiber mat; (**d**) SEM image of biofilm by *L. plantarum* GIM1.648 on (EC) nanofiber mat.

Cell growth and attachment are influenced by both the roughness as well as the hydrophobicity of a scaffold’s surface. For instance, Valamehr et al. reported that stem cells were more likely to proliferate and differentiate on hydrophobic surfaces [16], with hydrophobic interactions regarded as the dominant factor determining the magnitude of cell-surface adhesion [17]. In addition, it was found that a greater surface roughness promoted bacterial attachment by increasing the area of contact between the cells and the material surface [18], with the maximum reduction of bacterial adhesion on abutment surface occurring at a roughness of 0.2 μm [19]. Therefore, an average roughness above 0.2 μm is assumed to be favorable for biofilm formation. As far as the EC nanofiber mat prepared in this study was concerned, Figure 1a,b shows that the water contact angle and average roughness were 136° and 20 μm, respectively, thereby indicating its suitability for biofilm formation. Thus, it was used as a scaffold for biofilm formation by *L. plantarum* GIM1.648. After cultivation for 24 h, SEM was performed to observe the biofilm formed on the nanofibers. Compared with the control (Figure 1c), a high density of cells was found to be attached to the microfibers used as a scaffold (Figure 1d). In addition, a number of channels were present in the biofilm. These results, therefore, indicated that the nanofiber mat was an excellent scaffold material for biofilm formation by probiotics.

### 3.2. Effects of Media on Biofilm Formation

**Figure 2:**
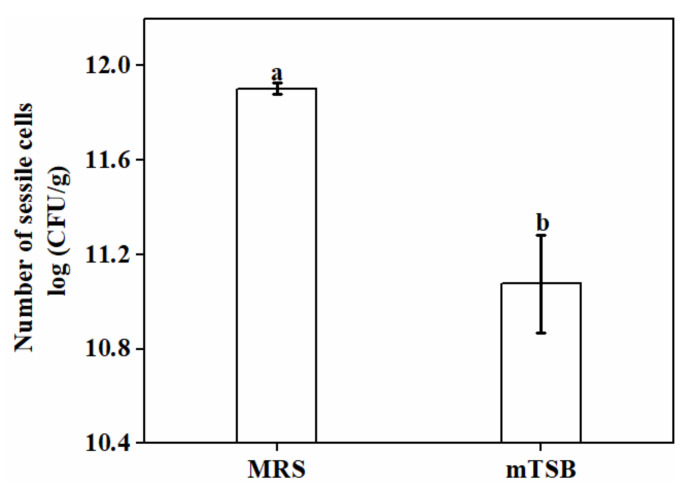
The effect of medium type on the number of sessile cells in biofilms. Significant differences (*p* < 0.05) are indicated by different letters (a and b).

MRS medium is often used for the isolation and culture of lactic acid bacteria (LAB), but modified trypticase soy broth (mTSB) has been reported as an optimal medium for its biofilm formation, despite its lack of salts and a lower concentration of carbon. Therefore, the ability of *L. plantarum* GIM1.648 to produce biofilms in the two media were compared under the same conditions (Figure 2). Overall, it was found that biofilm formation was less pronounced in mTSB medium compared to MRS (*p* < 0.05). Some authors reported that a lack of nutrients can increase the bacterial transition from a planktonic to a sessile mode [14]. The results obtained in this study seemed to disagree with this general behavior, since the number of sessile cells in the biofilm was greater in MRS medium. This could probably be attributed to differences between lactic acid bacteria strains.

**Figure 3:**
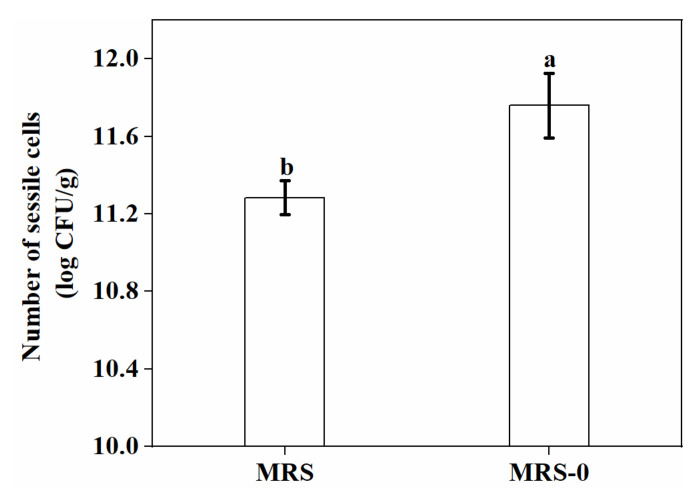
Effect of Tween 80 on the number of sessile cells in biofilms (MRS: with Tween 80; MRS-0: without Tween 80). Significant differences (*p* < 0.05) are indicated by different letters (a and b).

The nonionic surfactant Tween 80, as a component of the MRS medium, was previously reported to influence biofilm formation in some LAB, with MRS medium without the surfactant being more favorable for biofilm formation [20]. Hence, the capacity of *L. plantarum* GIM1.648 to form biofilms in the presence or absence of Tween 80 was compared. The results, presented in Figure 3, showed that biofilm formation was promoted in the absence of Tween 80 from MRS (*p* < 0.05). This observation was in agreement with the report by Terraf et al. [20]. The auto-aggregation (binding capability) level is an index used during the preliminary screening of bacterial capacity to produce biofilms. Ibarreche et al. [21] reported that a higher auto-aggregation level was achieved when MRS medium without Tween 80 was used, and this could have been due to the dispersing effects of Tween 80. Based on the above results, MRS medium without Tween 80 was selected for further experiments.

### 3.3. Impact of Culture Conditions on the Formation of Biofilms

**Figure 4:**
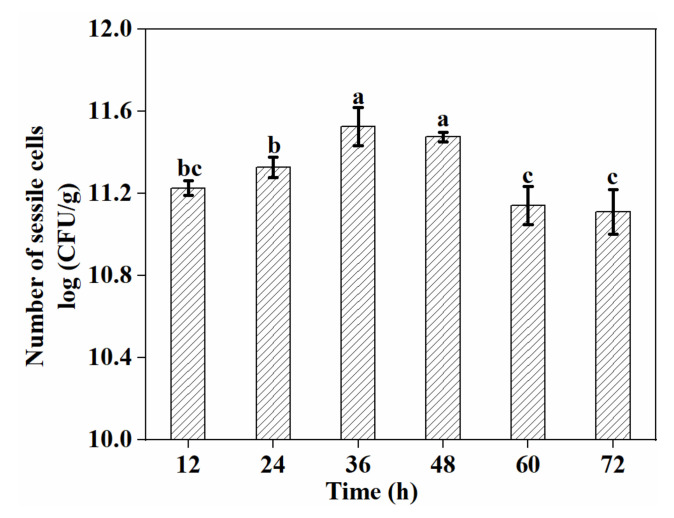
Impact of culture time on the number of sessile cells. Significant differences (*p* < 0.05) are indicated by different letters (a, b, and c).

Biofilm formation by *L. plantarum* GIM1.648 on the EC nanofiber mat was analyzed by CFU enumeration and SEM. As shown in Figure 4, culture time had an important impact on biofilm formation (*p* < 0.05), as the increasing length of incubation resulted in an increased number of sessile cells until a maximum was reached at 36 h. However, further increases in culture time decreased the number of sessile cells, with a decline of approximately 0.4 log CFU/g occurring after 60 h. This could have been because, as the culture time increased, not only were nutrients depleted, but the pH of the broth media also decreased, inhibiting bacterial growth. Furthermore, the limited spaces available on the porous nanofiber mats were being increasingly taken up by bacteria.

**Figure 5:**
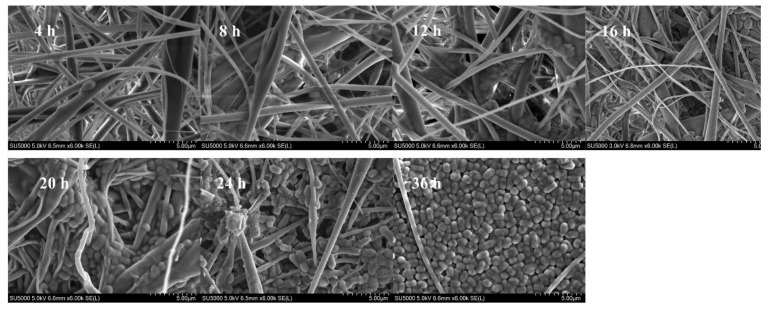
SEM images of biofilms formed on EC nanofiber mats by *L. plantarum* GIM1.648 at different culture times.

SEM was further applied to visualize the biofilms. Figure 5 shows that after 8 h, *L. plantarum* GIM1.648 occurred either individually or in pairs and was thus considered to be in a planktonic growth mode. After 12 h, some aggregation could be observed as cells formed bridges across fibers and created colonies using the substrate as a scaffold. Larger aggregates were then generated at 24 h, with many cells growing into the pores of the nanofiber mat, although gaps between cell aggregates remained clear. At 36 h, the cells almost completely occupied the mat, which was consistent with the above results obtained for CFU enumeration.

**Figure 6:**
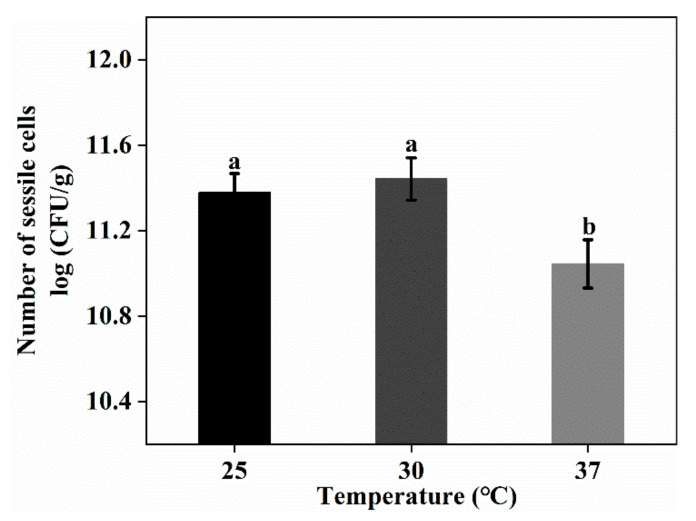
The influence of different culture temperatures on the number of sessile cells. Significant differences (*p* < 0.05) are indicated by different letters (a and b).

Temperature has a significant influence on the growth of bacteria as well as their ability to form biofilms. The effects of temperature on *L. plantarum*’s GIM1.648 ability to form biofilms were as shown in Figure 6. In this case, it was clear that, as the temperature increased from 25 °C to 30 °C, the number of sessile cells increased, with the optimal temperature for biofilm formation by *L. plantarum* GIM1.648 being 30 °C. Further increases in temperature up to 37 °C were not favorable for biofilm formation, although the latter is considered to be optimal for the growth of *L. plantarum* GIM1.648. These results were consistent with those reported in other studies [22,23]. It was also reported that the ability of different *L. rhamnosus* strains to form biofilms increased at lower growth temperatures [22]. For microorganisms other than LAB (e.g., *Staphylococcus aureus*), it was also found that the suboptimal growth temperature was more beneficial for biofilm formation [23]. This phenomenon could be explained by the fact that the expression level of genes related to biofilm formation is sensitive to the temperature [23]. In addition, bacterial biofilm is generally formed under stressful conditions [10].

**Figure 7:**
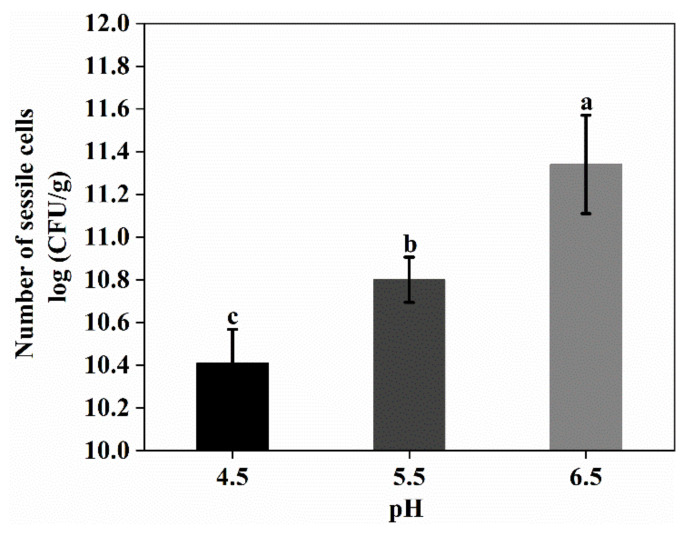
The effect of pH on the number of sessile cells. Significant differences (*p* < 0.05) are indicated by different letters (a, b, and c).

It has been reported that the biofilm formation of some lactic acid bacteria were enhanced in acidic media [24]. Thus, in this work, the effects of pH on biofilm formation by *L. plantarum* GIM1.648 were investigated. As shown in Figure 7, the selected strain could produce more biofilms with increasing pH. In this case, the number of sessile cells reached its maximum (11.34 log CFU/g) at pH 6.5, but this was in disagreement with the above-mentioned report [24]. In addition to differences in microbial strains, variations in experimental methods could account for the different results obtained in this study. Indeed, in the previous study, 96-well microtiter plates were used for biofilm formation, with the biofilms subsequently stained for quantification by spectrophotometry. On the other hand, this study used nanofiber mats as a scaffold for biofilm formation, and CFU enumeration was used to determine the number of sessile cells within the biofilms.

**Figure 8:**
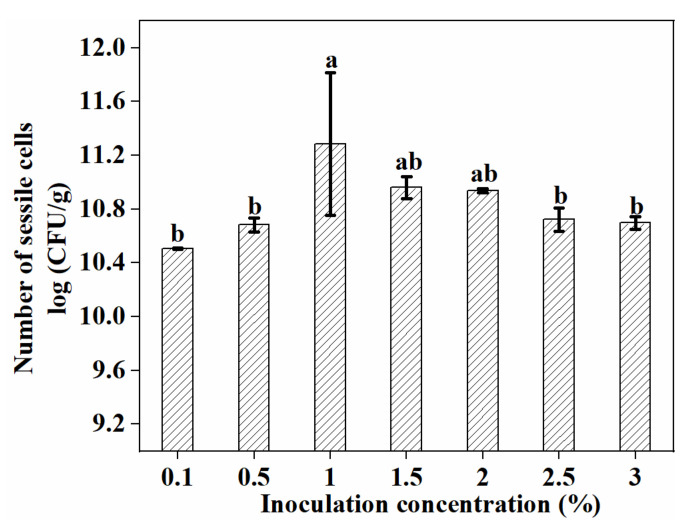
The effect of inoculation concentration on the number of sessile cells. Significant differences (*p* < 0.05) are indicated by different letters (a and b).

The influence of inoculum concentration on biofilm formation by *L. plantarum* GIM1.648 was as shown in Figure 8. Overall, the highest number of sessile cells was obtained at an inoculum concentration of 1% (*v/v*), with higher or lower concentrations decreasing their numbers. This result was in agreement with Hu’s report [7]. There was almost no statistically significant difference between 1% (*v/v*) and 2% (*v/v*) (*p* > 0.05). The possible reason may be that limited space in the fiber mat was remained for more cells to reside, as seen from Figure 5. Since inoculum 1% (*v/v*) was also suitable for the densest biofilm development, to save costs, it was used for further study. Altogether, based on the results, the optimal environmental conditions for biofilm formation by the selected strain was determined to be MRS medium without Tween 80, a culture time of 36 h, a culture temperature of 30 °C, pH 6.5, and an inoculum concentration of 1% (*v/v*).

### 3.4. Sessile Cells’ Survival against Environmental Stresses

**Figure 9:**
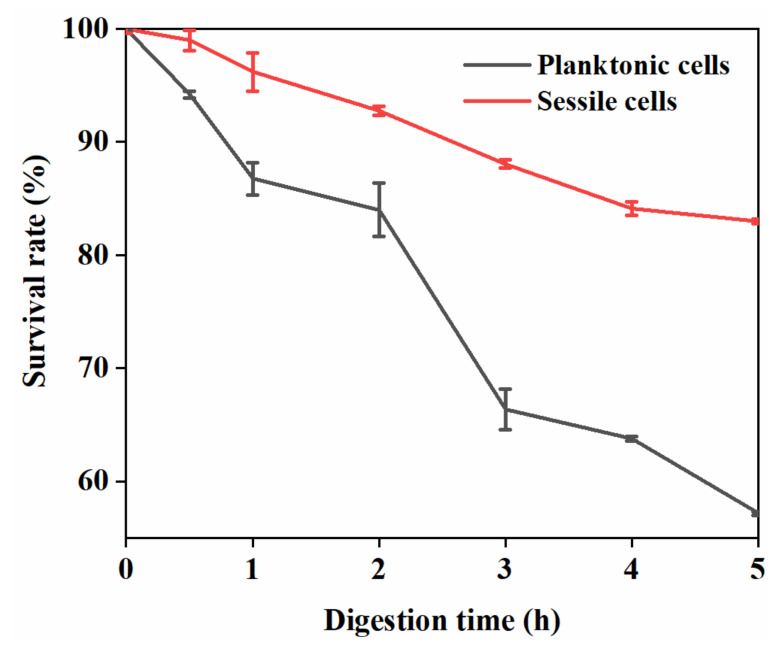
Survival rate of *L. plantarum* GIM1.648 in planktonic and biofilm modes after 5 h of digestion in a simulated gastrointestinal tract.

The suitability of probiotics is evaluated based on their ability to resist the damaging conditions of the GI tract [25]. Hence, in this study, the resistance of *L. plantarum*’s GIM1.648 sessile cells during in vitro digestion was explored. As shown in Figure 9, up to 92% of the cells in biofilm survived 2 h of gastric digestion, while the corresponding value for planktonic cells was only 83%. After digestion in a simulated GI tract for 5 h, 82% of the sessile cells were still viable, with this value representing only a 1.58 log CFU/g loss in viability. This was a clear improvement of 43.9% compared to planktonic cells (82% vs. 57%), indicating that the sessile cells in biofilms had better tolerance to GI tract conditions. He et al. also reported that some lactic acid bacteria had the ability to resist acid stress and display improved survivability in the GI tract after forming biofilms in microcapsules [9]. Hence, the formation of a biofilm is helpful to enhance the resistance of probiotics against gastrointestinal digestion.

**Figure 10:**
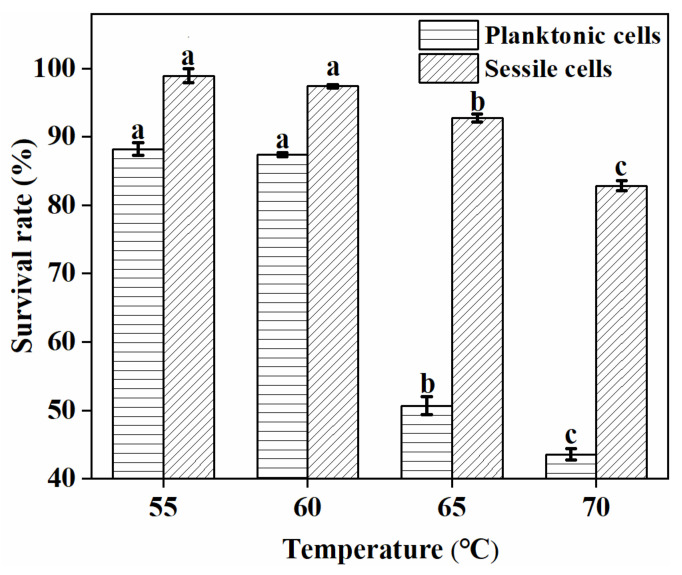
The effects of temperature on the viability of cells in planktonic and biofilm modes. Significant differences (*p* < 0.05) are indicated by different letters (a, b, and c).

It is crucial for probiotics to display heat resistance for their applications. In this study, the cells in planktonic and biofilm growth modes were treated at different temperatures in the range of 55–70 °C. As shown in Figure 10, treatments at 55 °C and 60 °C did not significantly influence the sessile cells’ viability (*p* > 0.05), while that of planktonic cells decreased to approximately 87%. Above 60 °C, there was a sharp decrease in the viability of planktonic cells, with only 43% surviving at 70 °C. In contrast, the sessile cells in biofilms displayed good heat stability, as their viability could reach over 82%, even at 70 °C. Cheow et al. [26] previously reported the superiority of biofilm over the planktonic cells in surviving harsh environments. Our results, while being similar to their findings, suggested that biofilm formation can improve the thermotolerance of probiotics.

### 3.5. luxS Expression

The *luxS* gene was a critical gene in the biofilm development of probiotic strains [6,10], and it has been shown that deletion of the *luxS* gene causes *L. rhamnosus* GG to fail in biofilm formation [27]. Lebeer et al. found that changes in cultivation conditions, such as temperature and medium, upregulated the expression of the *luxS* gene, thus enhancing the formation of biofilms [28]. Likewise, when over-expressed, the gene caused *Bifidobacterium longum* NCC 2705 to produce more biofilms [29]. In order to further understand the relationship between *luxS* expression and biofilm formation for *L. plantarum* GIM1.648, RT-qPCR was used to investigate changes in the *luxS* expression of planktonic and sessile cells.

**Figure 11:**
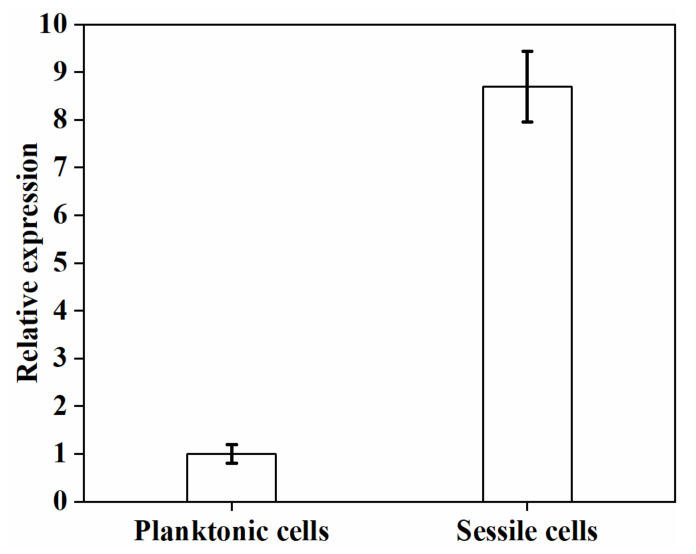
The expression level of *luxS* for sessile cells compared to planktonic cells.

As observed from Figure 11, the sessile cells in biofilms increased *luxS* expression, with the relative expression being 8.7-fold higher compared to planktonic ones. Previous reports found that the signal molecule autoinducer-2 (AI-2)’s synthesis was induced by *luxS*, and this could improve biofilm formation of lactic acid bacteria, including their ability to resist acids and bile salts [28,30]. These results clearly demonstrate that the *luxS* gene was involved in the biofilm formation by *L. plantarum* GIM1.648.

## 4. Conclusions

In this study, electrospun EC nanofiber mats, which can mimic the bionic structure of natural extracellular matrices, were applied as a scaffold for biofilm formation by *L. plantarum* GIM1.648. The water contact angle and average roughness of the prepared mat were 136° and 20 μm, respectively, and these factors were beneficial for biofilm formation. SEM results further confirmed that biofilms were indeed successfully formed on the fiber mats, with the optimal conditions for this process being MRS medium without Tween 80, a culture time of 36 h, a temperature of 30 °C, a pH of 6.5, and an inoculum concentration of 1% (*v/v*). By forming biofilms, the probiotic cells were also more resistant to environmental stresses. For instance, after digestion in a simulated GI tract for 5 h, 82% of sessile cells remained viable, and this represented an improvement of 43.9% compared to planktonic ones (82% vs. 57%). In addition, the sessile cells in biofilms exhibited greater thermal stability, as the survival rate was up to 83 ± 0.2%, even at 70 °C. When studying the relative expression level of the *luxS* gene, it was found that this gene was expressed 8.7-fold more in sessile cells compared to planktonic ones, thereby indicating that *luxS* was involved in the biofilm formation by *L. plantarum* GIM1.648. Altogether, the results of this study suggest that the formation of biofilm is an efficient strategy for improving the resistance of probiotics to adverse conditions. Furthermore, this work demonstrates that the electrospun nanofiber mat could be an excellent scaffold material for biofilm formation by probiotics, and this could further widen the potential applications of electrospun materials.

## Data Availability

Data are contained within the article.
